# Efficacy of an Unguided, Digital Single-Session Intervention for Internalizing Symptoms in Web-Based Workers: Randomized Controlled Trial

**DOI:** 10.2196/45411

**Published:** 2023-07-07

**Authors:** Lorenzo Lorenzo-Luaces, Jacqueline Howard

**Affiliations:** 1 Department of Psychological and Brain Sciences Indiana University-Bloomington Bloomington, IN United States; 2 TRAILS to Wellness Ann Arbor, MI United States

**Keywords:** internet-based cognitive behavioral therapy, iCBT, depression, transdiagnostic processes, emotion regulation

## Abstract

**Background:**

The Common Elements Toolbox (COMET) is an unguided digital single-session intervention (SSI) based on principles of cognitive behavioral therapy and positive psychology. Although unguided digital SSIs have shown promise in the treatment of youth psychopathology, the data are more mixed regarding their efficacy in adults.

**Objective:**

This study aimed to investigate the efficacy of COMET-SSI versus a waiting list control in depression and other transdiagnostic mental health outcomes for Prolific participants with a history of psychopathology.

**Methods:**

We conducted an investigator-blinded, preregistered randomized controlled trial comparing COMET-SSI (n=409) with an 8-week waiting list control (n=419). Participants were recruited from the web-based workspace Prolific and assessed for depression, anxiety, work and social functioning, psychological well-being, and emotion regulation at baseline and at 2, 4, and 8 weeks after the intervention. The main outcomes were short-term (2 weeks) and long-term (8 weeks) changes in depression and anxiety. The secondary outcomes were the 8-week changes in work and social functioning, well-being, and emotion regulation. Analyses were conducted according to the intent-to-treat principle with imputation, without imputation, and using a per-protocol sample. In addition, we conducted sensitivity analyses to identify inattentive responders.

**Results:**

The sample comprised 61.9% (513/828) of women, with a mean age of 35.75 (SD 11.93) years. Most participants (732/828, 88.3%) met the criteria for screening for depression or anxiety using at least one validated screening scale. A review of the text data suggested that adherence to the COMET-SSI was near perfect, there were very few inattentive respondents, and satisfaction with the intervention was high. However, despite being powered to detect small effects, there were negligible differences between the conditions in the various outcomes at the various time points, even when focusing on subsets of individuals with more severe symptoms.

**Conclusions:**

Our results do not support the use of the COMET-SSI in adult Prolific participants. Future work should explore alternate ways of intervening with paid web-based participants, including matching individuals to SSIs they may be most responsive to.

**Trial Registration:**

ClinicalTrials.gov NCT05379881, https://clinicaltrials.gov/ct2/show/NCT05379881

## Introduction

### Background

Internalizing symptoms are among the leading causes of disability in the United States and worldwide [1]. For example, major depression affects at least 20% of adults in the United States [2]. Although between individuals, depression is variable in its symptom presentation [3,4] and overall prognosis [5,6], at a societal level, it is a leading contributor to the global burden of disability [1]. Between individuals, depression strongly covaries with other internalizing symptoms (eg, generalized anxiety and emotional liability) as part of what has been called an *emotional dysfunction* superspectrum of symptoms that may reflect difficulties regulating negative emotions [7]. Depression is a major contributor to the emotional dysfunction superspectrum but other internalizing symptoms, such as anxiety, are similarly heterogeneous in presentation and prognosis [7].

Psychological interventions, the most widely studied of which are cognitive behavioral therapies (CBTs), target depression and other internalizing symptoms, partly by improving an individual’s capacity to regulate negative emotions, including increasing the use of cognitive reappraisal [8,9] and decreasing maladaptive avoidance strategies, such as suppressing the expression of emotions. These findings align with a meta-analysis of emotion regulation experiments [10], which reported that regulating emotions through cognitive reappraisal is associated with improvements in emotions (standardized mean difference [SMD] 0.45, 95% CI 0.35-0.56), and expressing emotions (ie, not suppressing their expression) is also associated with emotional outcomes (SMD 0.10, 95% CI 0.01-0.18).

It is widely recognized that current treatment efforts, which are mostly centered around individual interventions delivered by a professional, such as antidepressant medications and face-to-face psychological interventions such as CBTs, have not made a dent in the public health burden of depression and are unlikely to make a substantial public health contribution in the near future [11]. Prior studies have highlighted at least 2 strategies with the potential to reduce the public health burden of untreated depression. One strategy is to identify individuals who are at a relatively high risk of depression onset or relapse [12] or who experience health disparities. For example, cisgender women; lesbian, gay, bisexual, trans, queer, or other sexual minority (LGBTQ+) individuals; people with a prior history of depression; and individuals who are not in education, employment, or training are at high risk for depression onset and relapse after symptom improvement [12]. Individuals with identities that are minoritized based on race or ethnicity or who live in areas with limited access to health providers tend to have lower access to mental health services than their race or ethnic majority counterparts and individuals who live in areas with a high number of health providers [13].

Another strategy to decrease the public health burden of depression is to develop and disseminate interventions that are more scalable than antidepressants and individual face-to-face psychological interventions [11]. Digital mental health interventions (DMHIs) have emerged as some of the most popular treatment modalities because of the ubiquity of internet access. A substantial body of evidence suggests that DMHIs are effective when compared with waiting list controls (WLCs), care as usual, and other controls such as sham apps [11,14-16]. Digital single-session interventions (SSIs) have emerged as promising scalable treatments for internalizing symptoms in youth because SSIs are not time-consuming and can be made freely available [17,18]. In a digital SSI, an individual completes a web-based intervention in 1 sitting. Free-standing brief treatments, such as unguided digital SSIs, are also important interventions for the treatment of internalizing symptoms because engagement with face-to-face therapies and DMHIs in real-world settings is usual brief [17,18]. Meta-analyses in youth suggest that SSIs are more effective than control conditions, such as waiting lists, care as usual, or “placebo” SSIs [17,18].

In the treatment of adult psychopathology, individual face-to-face SSIs have a long history as treatments for specific phobias and obsessive-compulsive disorder [18], and other clinical problems are amenable to highly symptom-focused treatments. Unguided digital SSIs for the treatment of internalizing symptoms in adults have yielded mixed findings. For example, an instructor-led SSI for chronic pain appeared to be more effective than health psychoeducation and noninferior to an 8-week course of CBT [19]. However, in a meta-analysis by Schleider and Weisz [17], the effect sizes were very small for depression as a treatment target or secondary outcomes. Mullarkey et al [20] found no support for the efficacy of an unguided SSI focused on perceived control over anxiety versus a placebo control in a nationally representative sample of 500 adults. Thus, the efficacy of unguided digital SSIs for adult internalizing symptoms is still a matter to be established.

Wasil et al [21] developed an SSI called the Common Elements Toolbox (COMET) that captures elements common to CBT and positive psychology, including behavioral activation, cognitive restructuring, self-compassion, and gratitude. Adding positive psychology interventions to traditional CBT strategies may lead to especially positive outcomes because evidence suggests that interventions that target positive emotions may specifically target positive affect [22,23] or help individuals not helped by traditional CBT [24]. In an initial investigation, undergoing COMET was associated with improvements in perceived coping abilities, and the intervention was feasible and acceptable to graduate students [21]. In the first randomized controlled trial (RCT) evaluating the efficacy of COMET, Steinberg et al [25] reported that COMET reduced depression in undergraduate students when compared with a study skills control condition at 4-week (SMD 0.20) and 12-week follow-ups (SMD 0.17). Nonetheless, these small effects may be promising for scalable treatments, such as unguided digital SSIs.

Given the high potential for dissemination of a free SSI such as COMET and the preliminary evidence for its efficacy, we studied the efficacy of COMET in a population at high risk for depression: paid web-based participants. Researchers are the most familiar with paid web-based participants because they often use them in survey studies and web-based experiments [26]. Existing data suggest that paid web-based participants report very high levels of depression when compared with the general population [27], although somewhat lower than other convenience samples, such as college students [28]. Ophir et al [27] showed that only some of this elevated depression is attributable to inattentive or potentially fraudulent respondents (eg, bots). Their results suggest that sociodemographic characteristics, especially the level of physical activity and sedentary behavior, explain some of the risk for depression associated with web-based work, but most of the variance in the risk for depression in paid web-based participants versus the general population remains unexplained. McCredie and Morey [29] characterized paid web-based participants as being more neurotic and less socially engaged than the rest of the population. This suggests that paid web-based participants are at an elevated risk for depression and have documented risk factors for psychopathology that may ameliorate using psychological interventions such as COMET.

Prior studies suggest that intervention trials with paid web-based participants are feasible. For example, in a factorial RCT exploring variations of cognitive bias modification, Steinman et al [30] recruited 1120 individuals with moderate to severe anxiety and treated them with variations of cognitive bias modification. The results suggested that some variations of cognitive bias modification appeared slightly better at manipulating anxiety-related cognitive biases, but the study did not include a postbaseline assessment exploring the intervention’s effect on anxiety or depression (ie, the study focused on the engagement of cognitive biases with treatment). Mullarkey et al [20] attributed their failure to find statistically significant intervention effects on the characteristics of their sample (ie, recruiting healthy adults vs clinical high-risk groups), their outcome choices (ie, anxiety and perceived control), and the use of active control, which may have limited the finding of small effects.

### Objectives

We conducted an RCT to evaluate the short-term (2 weeks) and long-term (8 weeks) efficacy of the COMET-SSI in paid web-based participants, addressing the limitations of prior studies on SSIs in adult paid web-based participants [20]. We focused on adults with ongoing or previous mental health problems who may be at risk for current symptoms; assessed a wide variety of outcomes, including internalizing symptoms, emotion regulation, and functioning; and used a WLC. We chose to study COMET-SSI given its feasibility and preliminary evidence for its efficacy. We hypothesized that the COMET would lead to small improvements in depression, anxiety, well-being, functioning, and emotion regulation.

## Methods

### Overview

This was an RCT that has a preregistration for its design (ClinicalTrials.gov NCT05379881) and analysis plan [31]. It was a 2-arm parallel RCT with a 1:1 allocation ratio, comparing short-term (ie, 2 weeks) and long-term (ie, 8 weeks) depression and anxiety changes in paid web-based participants randomized to receive the intervention COMET or a WLC. This trial aimed to assess the efficacy of COMET on primary and secondary outcomes. We determined the choice of control condition using the Pragmatic Model for Comparator Selection in Health-Related Behavioral Trials [32,33]. This framework specifies that no one control group is optimal, but the primary aim of the trial and limitations and barriers must be considered when choosing a control condition. Although some control groups, such as placebo controls, tend to be more methodologically rigorous than other controls, they may not be the best control group in any specific study. One consideration in designing our trial was that the efficacy of SSIs, in general, has been well-established in youth [17]. Although meta-analyses suggest that the effects of psychological interventions are stronger in adults than in youth [15], the efficacy of SSIs in adults and of the COMET specifically is not as well-established as the efficacy of SSIs in youth. Thus, we chose to compare the COMET with a WLC.

### Participants

Paid web-based participants living in the United States were sampled from Prolific [34], a web-based platform hosting research studies. The only inclusion criterion we used for the trial was an affirmative answer (ie, responding “yes”) to the question “Do you have—or have you had—a diagnosed, ongoing mental health/illness/condition?” On the basis of this inclusion criterion and sampling from the United States, 14,628 individuals were eligible for the study. Participants were informed that this was an Indiana University study that explored the efficacy of the COMET. No explicit exclusion criteria were applied. However, it could be argued that relatively steady internet access and internet literacy were entry criteria because the study required individuals to log in to Prolific 1 to 4 times.

### Ethics Approval and Participation

This study (#12290) was approved by the human subject research ethics review board of Indiana University. The study was described to participants as “testing the efficacy of an online wellness activity called the Common Elements Toolbox (COMET) [which] helps you learn skills that may help to improve your mood and well-being.” Participants were paid a maximum of US $12.02 for participation in the study, a rate of roughly US $8 an hour. This was divided into payments of US $8 for the baseline assessment, irrespective of treatment condition, and 3 payments of US $1.34 for the 2-, 4-, and 8-week surveys. The participants were assured that their study data were deidentified.

### Randomization

The study was hosted in Qualtrics (Qualtrics International Inc), and randomization was performed within the Qualtrics platform, which uses simple randomization. Thus, the treatment allocation schedule was concealed from the investigator and the participants. A study coordinator coded the treatment condition, concealing the coding scheme from the principal investigator (PI). To prepare all analyses associated with this study, the PI used the R (R Foundation for Statistical Computing) [35] programming language to create a synthetic treatment variable that had no correspondence to the actual treatment assignment. Owing to the nature of the intervention, the actual treatment assignment could not be concealed from participants, who were the ones who assessed outcomes.

### Sample Size and Power

An a priori analysis in G*Power (version 3.1; Heinrich-Heine-Universität Düsseldorf) [36] suggested that to have 80% power to detect a small treatment effect (SMD 0.20) using a *P* value of <.05 as the cutoff and with 1:1 allocation, we would need to recruit 788 individuals. We did not factor in attrition because we planned to impute data but aimed to recruit 800 individuals because we had available funding for slightly more than 788 individuals. We posted the study to Prolific on July 5, 2022, and enrolled 800 individuals on the same day. We accepted and compensated participants for the study if they completed the baseline battery of assessments, which would take approximately 15 minutes. As Prolific only counts participants as completing a study if they scroll all the way through to the end, we recruited 861 individuals. Of these 861 individuals, 33 (3.8%) did not complete the baseline survey; therefore, only 828 (96.1%) participants were randomized.

### Allocation Groups

#### WLC Group

Participants assigned to the WLC filled out the same assessment battery as participants assigned to the COMET but did not have access to the COMET until 8 weeks after the study. The outcomes were not tracked after the 8-week assessment period. As the Prolific platform requires a 1-time estimate for the study as opposed to allowing different time estimates per condition, we attempted to make the groups somewhat more equal to the amount of time spent in the study by administering participants in the WLC with additional questions assessing how important changes in depression and anxiety were to them. Specifically, once participants were assigned to the WLC, they were once again presented with the items on the Patient Health Questionnaire (PHQ)-9 and Generalized Anxiety Disorder-7 (GAD-7), and for each of the 15 items, they were asked to rate (1) how important of concerning the symptom is, if they had it, or would be if they developed it; (2) how detrimental to subjective quality of life it is or would be; (3) how detrimental to objective functioning it is or would be; and (4) how much they would want the symptom to improve.

#### COMET Group

The COMET is a self-guided and unguided 4-module intervention designed based on 2 core principles of CBT, cognitive restructuring and behavioral activation, and 2 core principles of positive psychology, gratitude and self-compassion. The COMET was designed to take approximately 30 to 40 minutes to complete. It presents individuals with psychoeducation in the form of texts and brief exercises. Specifically, participants were shown the “ABCD” technique (for cognitive restructuring), scheduling pleasant activities (for behavioral activation), the “three good things” exercise (for gratitude), and writing self-compassionate statements (for self-compassion). Previous studies supporting the efficacy of COMET include an open trial [21] and an RCT [25]. Participants were advised that “[t]o get the most out of these skills, many people find it useful to practice them regularly.” We recommended that participants practice at least one of the skills daily for the succeeding 2-week period. However, there were no formal reminders regarding skill use.

### Measures

All measures were self-reported web-based assessments administered at baseline (ie, July 5, 2022), two weeks after baseline (July 19 to 21, 2022), four weeks after baseline (August 2 to 4, 2022), and 8 weeks after baseline (August 30 to September 3, 2022). One deviation from the trial protocol is that a day before the end of the 8-week assessment, we reminded participants in either treatment condition who had not yet completed the intervention that the study assessments were available in Prolific.

### Primary Outcomes

#### PHQ Score

The PHQ-8 is a subset of the PHQ-9 removing the question of death ideation and self-injury. The PHQ-9 [37] is a 9-item self-report questionnaire that assesses the frequency of Diagnostic and Statistical Manual of Mental Illnesses symptoms of a major depressive episode. The scale was developed as a screening tool for primary care. Responses range from 0 (“not at all”) to 3 (“nearly every day”), producing scores from 0 to 27, with higher scores indicating more frequent depressive symptoms. We administered the PHQ-9 minus the item assessing recurrent thoughts of death or thoughts of self-harm (ie, we administered the PHQ-8). The PHQ-8 appeared to be an internally consistent measure of depressive symptoms at baseline (*ω*=0.88, 95% CI 0.87-0.89), week 2 (*ω*=0.88, 95% CI 0.87-0.90), week 4 (*ω*=0.89, 95% CI 0.88-0.91), and week 8 (*ω*=0.88, 95% CI 0.87-0.89). We prorated the PHQ-8 scores by multiplying them by 1.125, so they would be on a scale of 0 to 27, similar the PHQ-9 metric. A score ≥10 was considered a positive screen for major depression [37].

#### GAD-7 Score

The GAD-7 [38] is a 7-item self-report questionnaire that assesses the frequency of anxiety symptoms. It was developed as a screening tool for primary care. Responses range from 0 (“not at all”) to 3 (“nearly every day”), producing scores from 0 to 21, with higher scores indicating more frequent depressive symptoms. GAD-7 appeared to be an internally consistent measure of generalized anxiety at baseline (*ω*=0.91, 95% CI 0.90-0.92), week 2 (*ω*=0.91, 95% CI 0.90-0.92), week 4 (*ω*=0.91, 95% CI 0.90-0.92), and week 8 (*ω*=0.91, 95% CI 0.90-0.92). A score ≥10 was considered a positive screen for anxiety [38].

### Secondary Outcomes

#### World Health Organization Well-Being Index

The World Health Organization Well-Being Index-5 (WHO-5) [39] is a 5-item self-report scale that measures subjective well-being, an aspect of positive mental health. Its items are rated on a scale of 0 (“at no time”) to 5 (“all the time”). The raw total scores (range 0-25) are multiplied by 4, producing final scores ranging from 0 to 100, where higher scores indicate greater well-being. Prior work supports the reliability and validity of the WHO-5 [39], and it has previously been studied as an outcome measure in CBT self-help studies [40]. A score of 50 is considered a useful cutoff for screening for major depression. In this study, the WHO-5 appeared to be an internally consistent measure of well-being at baseline (*ω*=0.90, 95% CI 0.89-0.91), week 2 (*ω*=0.91, 95% CI 0.90-0.92), week 4 (*ω*=0.92, 95% CI 0.90-0.93), and week 8 (*ω*=0.91, 95% CI 0.90-0.92). A score of ≤50 is considered indicative of low well-being and has been used as a useful cutoff for screening for major depression [39].

#### Work and Social Adjustment Scale

The Work and Social Adjustment Scale (WSAS) [41] is a 5-item self-report measure that assesses impairment in work, relationships, household, and leisure activities because of a specific problem. As depression is a part of a broader set of internalizing symptoms, in our study, we queried the effect of the more colloquial term “stress” on functioning (eg, “[b]ecause of my *stress* my ability to form and maintain close relationships with others, including those I live with, is impaired”). Each item is rated on a 9-point Likert scale, ranging from 0 (“not at all”) to 8 (“very severely”), producing scores ranging from 0 to 40, with higher scores indicating greater impairment. Scores of >10 and >20 were considered to indicate moderate and severe impairment, respectively. Prior work supports the reliability and validity of the WSAS across various patient populations [42]. The WSAS appeared to be an internally consistent measure of work and social adjustment at baseline (*ω*=0.89, 95% CI 0.87-0.90), week 2 (*ω*=0.92, 95% CI 0.90-0.93), week 4 (*ω*=0.93, 95% CI 0.92-0.94), and week 8 (*ω*=0.92, 95% CI 0.91-0.93). A score ≥20 has been identified as signaling moderate impairment [41].

#### Emotion Regulation Scale

The Emotion Regulation Questionnaire (ERQ) [43] is a 10-item self-report measure of individual differences in the use of 2 emotion regulation strategies: cognitive reappraisal (ERQ-reappraisal; items 1, 3, 5, 6, 8, and 10) and expressive suppression (ERQ-suppression; items 2, 4, 6, and 9). Prior work supports the reliability and validity of the ERQ in community samples [44,45]. The ERQ items are rated on a 7-point Likert scale, with responses ranging from 1 (“strongly disagree”) to 7 (“strongly agree”). We averaged item scores to produce final scores on the same metric as the original items (ie, 1-7) to make the ERQ-reappraisal and ER-suppression subscales (with differing numbers of items) comparable. In this study, the baseline scores on the ERQ-reappraisal scale appeared to be an internally consistent measure of reappraisal at baseline (*ω*=0.91, 95% CI 0.89-0.92), week 2 (*ω*=0.93, 95% CI 0.92-0.94), week 4 (*ω*=0.93, 95% CI 0.92-0.94), and week 8 (*ω*=0.92, 95% CI 0.91-0.94). ERQ-suppression appeared to be an internally consistent measure of suppression at baseline (*ω*=0.82, 95% CI 0.79-0.84), week 2 (*ω*=0.83, 95% CI 0.80-0.85), week 4 (*ω*=0.85, 95% CI 0.83-0.87), and week 8 (*ω*=0.86, 95% CI 0.84-0.88).

### Satisfaction Data

At the request of a reviewer, we have included additional data attesting to treatment satisfaction with the COMET. At the end of the intervention, participants (n=381) were queried about their satisfaction with the intervention with questions about the program in terms of (1) their approval of it, (2) the degree to which they found it appealing, (3) their satisfaction with it, and (4) their willingness to integrate it into their lives. Responses were provided on a 4-point scale, ranging from 0 to 4. We calculated the mean and SD of the 4 questions. Responses to these questions appeared to be internally consistent indicators of satisfaction with the intervention (*ω*=0.96, 95% CI 0.94-0.97).

### Inattention

Prior studies suggest that inattentive or potentially fraudulent respondents may provide biased data. Following best practices in the detection of inattentive or fraudulent respondents, we used a multimethod approach to identify inattentive or potentially fraudulent respondents. First, we obtained an estimate of the time it took the participants to complete the baseline portion of the study. Given that the treatment condition would be a significant predictor of the time spent on the study at baseline, we *z*-scored the duration for each participant within each treatment condition. Respondents taking <2.5 SDs to complete the first part of the study were deemed as completing the study implausibly fast. In addition, at baseline, we administered the 10-item Personality Inventory [46], a measure of the Big Five personality traits. As the 10-item Personality Inventory contains 5 items that are scored in one direction (per subscale) and 5 items that are calculated in another direction, we were able to capture response inconsistency on this scale. We considered respondents as “inconsistent” if >40% of their responses were in an inconsistent direction. We also used RelevantID as a third attention check and the reCaptcha score as a fourth attention check [47]. RelevantID is a proprietary algorithm that analyzes the user’s browser, operating system, and location to provide a fraud score ranging from 0 to 100, with scores >30 usually understood as bots. The reCaptcha scores range from 0 to 1, with a higher score indicating a higher likelihood that the respondent is human. Scores of ≥0.5 are generally considered human responders.

### Analysis Plan

#### Overview

All analyses were conducted using the R programming language in the R Studio graphical user interface. We relied on *tidyverse* language for most data manipulation [48]. The analytic plan was preregistered on the Open Science Foundation website, which also contains the data and code that produced this manuscript [31,49]. The only deviation from the preregistered plan was that when the models failed to converge, we dropped the random slopes of time for a more parsimonious model. This occurred when examining changes in WHO-5 and ERQ-suppression in the intent-to-treat (ITT)–unimputed samples and WSAS in the ITT-imputed samples. For these analyses, we simplified the models by removing the random slopes, which allowed the models to converge.

The analyses were first constructed by concealing the treatment condition from the PI (LL-L) using a synthetic treatment condition variable (ie, a variable that randomly declared individuals as assigned to treatment vs control but did not correspond to the Qualtrics treatment assignment). First, we report the descriptive statistics, overall and by treatment. For categorical variables, we reported the frequency and percentage of each assessed value. For continuous variables, we reported the means and SDs. Consistent with published guidelines, we assumed that the randomization was effective and did not conduct tests to explore differences between the treatment conditions [50].

For all outcomes (ie, PHQ-9, GAD-7, WHO-5, WSAS, ERQ-reappraisal, and ERQ-suppression), we used linear mixed models with restricted maximum likelihood in *lme4* [51] and *lmerTest* [52] functions to regress outcomes on time (as a factor representing time 0, two weeks after baseline, 4 weeks after baseline, and 8 weeks after baseline), treatment (WLC vs COMET), and the time-by-treatment interaction.

The primary outcomes were depression (PHQ-9) and anxiety (GAD-7) changes at 2 weeks, which were given by the interaction of treatment-by-week 2, and 8 weeks, given by the treatment-by-week 8 interaction. The secondary outcomes were the 8-week changes in the WHO-5, WSAS, ERQ-CR, and ERQ-ES, which were given by the interaction of treatment with week 8. Standardized effect sizes were calculated using the method suggested by Feignhold [53], which involves dividing the estimated difference between treatment and control at different time points by the SD of the baseline assessment on each measure. To calculate the 95% CI on these d-type effect sizes, we used the *cohen.d.ci* function in the *psych* package [54].

Additional packages that were used include *labelled* to output variable names and *gtsummary* and *flextables* to create tables.

#### Missing Data Imputation

Missing data in demographic covariates were minimal (see the *Results* section), with most individuals providing complete information for most variables. More data were missing for the longitudinal outcomes (ie, PHQ-9, GAD-7, WHO-5, WSAS, and ERQ). To address missing data, including missing outcome data, we imputed all missing data using a machine learning algorithm: nonparametric missing value imputation using random forests (500 trees), with the R package *missForest* [55]. To preserve the association between the variables [56], we did not preprocess the variables and only minimally recoded them. Imputation models that use multiple variables are often preferable to the last observation carried forward-type imputation models [57,58]. The variables in the imputation model included baseline demographics and weeks 2, 4, and 8 scores on the PHQ-9, GAD-7, WHO-5, WSAS, and ERQ.

The analytical plan was repeated 3 different times. The first time followed an ITT approach with all individuals who were randomized and without imputed any outcomes (ITT-unimputed). The second time, an ITT approach was used, but missing outcome data were imputed as described earlier (ITT-imputed). Once the results with the ITT-unimputed and ITT-imputed data were clear, the PI was “unblinded” from the treatment assignment to construct the third sample: the “per-protocol” sample. The per-protocol sample included individuals only if they followed the intervention completely to the end (ie, they were not just randomized but completed the condition they were assigned to).

## Results

### Sociodemographics

**Figure 1:**
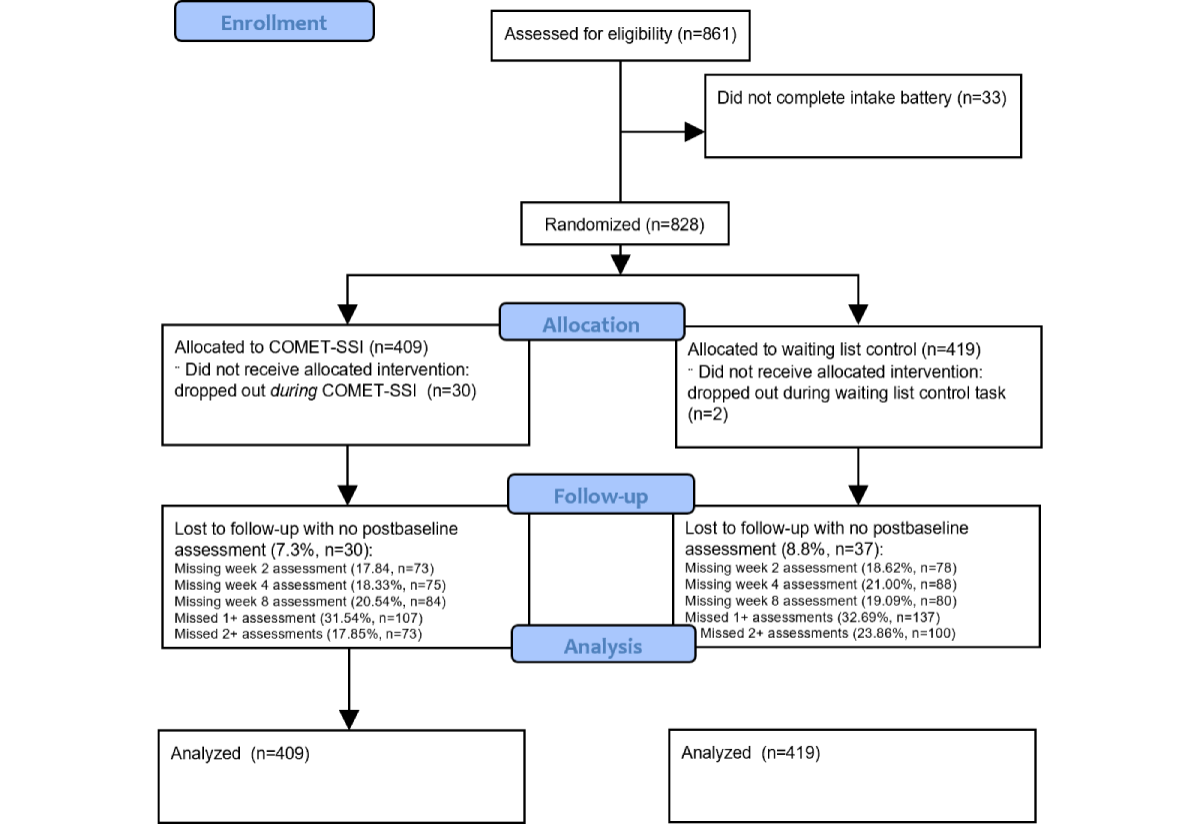
CONSORT (Consolidated Standards of Reporting Trials) 2010 flow diagram describing participant progression through a randomized controlled trial of a single-session intervention (SSI) versus waiting list control. COMET: Common Elements Toolbox.

A total of 861 individuals began the study, but 33 (3.8%) did not complete the baseline screening (Figure 1). Most participants met at least one cutoff of clinical significance in the PHQ-9, GAD-7, WSAS, or WHO-5 (732/828, 88.4%), with a median of 2 cutoff values being met. Of all individuals who completed the baseline screening (n=828), a total of 479 (57.6%) met the PHQ-9 screening for major depression and 348 (42%) met the cutoff score for generalized anxiety on the GAD-7. In total, 616 (74.4%) individuals met the WHO-5 screening criteria for major depression. More than half (n=421, 50.9%) of the participants met the WSAS criteria for poor functioning, and 83.3% (n=690) had a lifetime history of antidepressant use. On average, the participants were in their mid-30s, although they reported an early age of onset of symptoms of depression, anxiety, or stress. The female-to-male sex assigned at birth ratio was roughly 2:1. Approximately one-third (n=280, 33.8%) of the participants identified as LGBTQ+. The other demographics are presented in Table 1. No harms were reported by participants.

**Table 1:** Sociodemographic and clinical characteristics for 828 paid web-based participants randomized 1:1 to the Common Elements Toolbox versus a waiting list control group.

Characteristics		Waiting list control (n=419)	Common Elements Toolbox–single-session intervention (n=409)
**Age (years), mean (SD)**		36.01 (12.01)	35.46 (11.8)
	Unknown, n (%)	1 (0.2)	1 (0.2)
**Age of onset of internalizing symptoms (years), mean (SD)**		13.75 (7.8)	13.29 (6.8)
	Unknown, n (%)	2 (0.5)	0 (0)
**Sex assigned at birth, n (%)**			
	Male	138 (32.9)	140 (34.2)
	Female	277 (66.1)	267 (65.3)
	Intersex, inconclusive, or other	0 (0)	1 (0.2)
	Unknown	4 (0.9)	1 (0.2)
**Race and ethnicity, n (%)**			
	Black	30 (7.2)	21 (5.1)
	Hispanic	42 (10)	27 (6.6)
	White	294 (70.2)	312 (76.3)
	Other	44 (10.5)	44 (10.8)
	Unknown	9 (2.2)	5 (1.2)
**Sexual orientation, n (%)**			
	Heterosexual or straight	280 (66.8)	260 (63.6)
	Lesbian, gay, bisexual, trans, queer, or other sexual minority	134 (32)	146 (35.7)
	Unknown	5 (1.2)	3 (0.7)
**Marital status, n (%)**			
	Married or dating	169 (40.3)	190 (46.5)
	Single, divorced, or widowed	249 (59.4)	219 (53.5)
	Unknown	1 (0.2)	0 (0)
**Educational attainment, n (%)**			
	Less than high school	1 (0.2)	7 (1.7)
	High school diploma or equivalent (eg, General Educational Development)	61 (14.6)	50 (12.2)
	Some college	104 (24.8)	126 (30.8)
	Associate’s degree	48 (11.5)	46 (11.3)
	Bachelor’s degree	146 (34.8)	123 (30.1)
	Master’s degree	49 (11.7)	48 (11.7)
	Doctoral or professional degree	8 (1.9)	9 (2.2)
	Unknown	2 (0.5)	0 (0)
**Yearly income (US $), n (%)**			
	<15,000	38 (9.1)	42 (10.3)
	<15,000-24,999	38 (9.1)	39 (9.5)
	<25,000-34,999	55 (13.1)	44 (10.8)
	<35,000-49,999	54 (12.9)	79 (19.3)
	<50,000-74,999	86 (20.5)	80 (19.6)
	<75,000-99,999	53 (12.6)	55 (13.4)
	<100,000-149,999	45 (10.7)	39 (9.5)
	<150,000-199,999	28 (6.7)	13 (3.2)
	>200,000	10 (2.4)	8 (2)
	Unknown	12 (2.9)	10 (2.4)
**Employment status, n (%)**			
	Employed full time	197 (47)	181 (44.3)
	Employed part time	70 (16.7)	86 (21)
	Student	22 (5.2)	32 (7.8)
	Unemployed	129 (30.8)	110 (26.9)
	Unknown	1 (0.2)	0 (0)
**Antidepressant medication use, n (%)**		356 (85)	334 (81.7)
	Unknown	1 (0.2)	1 (0.2)
**Alcohol use, n (%)**			
	Never	126 (30.1)	128 (31.3)
	Monthly or less	118 (28.2)	145 (35.4)
	2-4 times a month	87 (20.8)	70 (17.1)
	2-3 times a week	51 (12.2)	43 (10.5)
	4+ times a week	36 (8.6)	23 (5.6)
	Unknown	1 (0.2)	0 (0)
**Depression (Patient Health Questionnaire-9; range 0-27), mean (SD)**		10.96 (6.64)	12.12 (6.76)
	Unknown, n (%)	1 (0.2)	1 (0.2)
**Anxiety (Generalized Anxiety Disorder-7; range 0-21), mean (SD)**		9.08 (5.50)	9.04 (5.45)
	Unknown, n (%)	2 (0.5)	0 (0)
Well-being (World Health Organization Well-Being Index-5; range 0-100), mean (SD)		37.88 (21.53)	34.02 (21.32)
**Functioning (Work and Social Adjustment Scale; range 0-40), mean (SD)**		18.93 (10.11)	19.74 (10.56)
	Unknown, n (%)	0 (0)	1 (0.2)
Cognitive reappraisal (ERQ^a^; range 1-7), mean (SD)		4.54 (1.25)	4.42 (1.30)
Expressive suppression (ERQ; range 1-7), mean (SD)		3.76 (1.47)	3.92 (1.50)

^a^ERQ: Emotion Regulation Questionnaire.

### Engagement

There were statistically significant differences (*χ*^2^_1_=24.3, *P*<.001) between the treatment conditions in the rate of completing the allocated intervention. Individuals in the WLC were more likely to complete the baseline rating task (417/419, 99.5%) than individuals in the COMET were to complete the COMET-SSI (379/409, 92.7%); however, these differences were small. There were no statistically significant differences between the treatment conditions in the rate of return for the 2-week assessment from July 19 to 21, 2022 (*χ*^2^_1_=0, *P*=.99); 4-week assessment from August 2 to 4, 2022 (*χ*^2^_1_=1.1, *P*=.29); or 8-week assessment from August 30 to September 3, 2022 (*χ*^2^_1_=0.2, *P*=.66).

A few individuals were flagged with 1 inattention check (n=40), and only 1 person was flagged with 2 inattention checks. The differences in the rate of inattentive respondents between the treatment conditions were not statistically significant (*P*=.07). At the request of a reviewer, we reviewed all text responses to the COMET and coded and rated whether they appeared to be valid responses to the prompts (eg, if individuals responded with activities to the behavioral activation module). Of the individuals who reached the first activity (n=408), behavioral activation or “pleasant activities,” all provided answers that were deemed valid responses. Of the individuals who reached the point of cognitive restructuring or “flexible thinking” (n=388), all but 3 (99.2%) individuals provided valid answers. Similarly, of the 388 individuals who reached the “gratitude” component, all but 3 (99.2%) provided valid answers. Of the 381 individuals who reached the final module, self-compassion, 379 (99.5%) provided valid answers. Thus, most individuals appropriately engaged with the web-based content.

### ITT-Imputed

**Figure 2:**
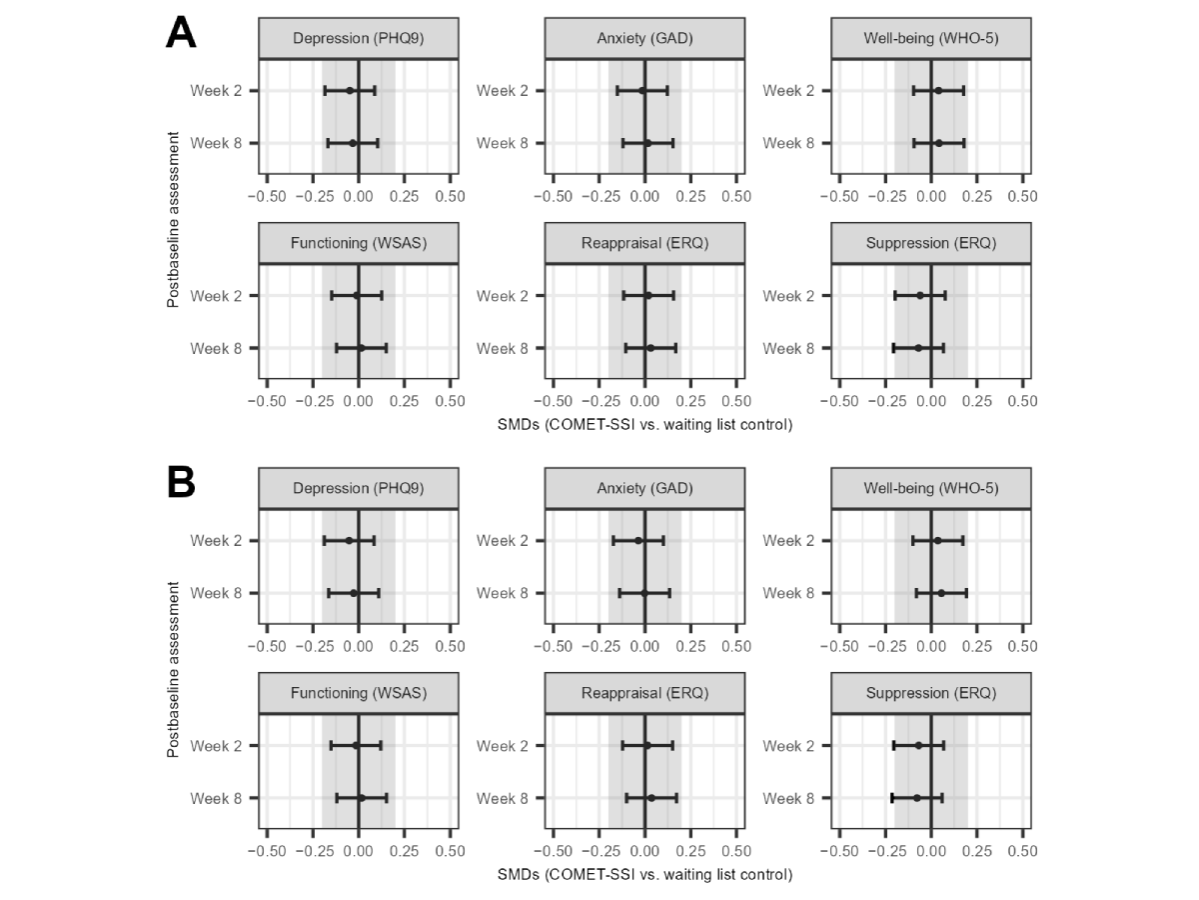
Standardized mean differences between the Common Elements Toolbox single-session intervention (COMET-SSI) and a waiting list control in the imputed (A) and unimputed (B) data (n=828). ERQ: Emotion Regulation Questionnaire; GAD: Generalized Anxiety Disorder; PHQ-9: Patient Health Questionnaire-9; WHO-5: World Health Organization Well-Being Index-5; WSAS: Work and Social Adjustment Scale.

Overall, there were no statistically significant differences between the COMET (n=409) and WLC (n=419) in the short-term primary outcomes of depression and anxiety (Table 2). Not only were outcomes not significantly different between the groups but also the magnitude of the differences was quite small (Figure 2A). Similarly, there were no statistically significant differences between treatments in terms of well-being, psychosocial functioning, or emotion regulation. The 8-week data showed an almost identical pattern. In other words, there were no statistically significant differences between the treatment conditions.

**Table 2:** Changes over time in depression, anxiety, well-being, functioning, and emotion regulation in 828 individuals in a randomized controlled trial of a single-session intervention (n=409) versus a waiting list control (n=419), imputation sample.

Characteristics		Depression (Patient Health Questionnaire-9), B (95% CI)	Anxiety (Generalized Anxiety Disorder-7), B (95% CI)	Well-being (World Health Organization Well-Being Index-5), B (95% CI)	Functioning (Work and Social Adjustment Scale), B (95% CI)	Reappraisal (Emotion Regulation Questionnaire), B (95% CI)	Suppression (Emotion Regulation Questionnaire), B (95% CI)
Intercept		11.0 (10.3 to 11.6)	9.09 (8.57 to 9.60)	37.9 (35.8 to 40.0)	18.9 (17.9 to 19.9)	4.54 (4.42 to 4.66)	3.76 (3.62 to 3.90)
**Time**							
	Week 0	—^a^	—	—	—	—	—
	Week 2	−0.95 (−1.28 to −0.62)	−0.96 (−1.25 to −0.67)	1.88 (0.50 to 3.26)	−1.79 (−2.38 to −1.20)	0.07 (0.00 to 0.14)	−0.02 (−0.10 to 0.05)
	Week 4	−0.90 (−1.26 to −0.54)	−1.06 (−1.37 to −0.75)	1.51 (0.02 to 3.00)	−1.62 (−2.25 to −0.99)	0.00 (−0.08 to 0.07)	−0.08 (−0.16 to 0.00)
	Week 8	−1.37 (−1.78 to −0.96)	−1.31 (−1.65 to −0.97)	2.97 (1.31 to 4.63)	−1.95 (−2.64 to −1.26)	0.10 (0.02 to 0.18)	−0.03 (−0.11 to 0.06)
**Treatment**							
	Waiting list control	—	—	—	—	—	—
	COMET-SSI^b^	1.16 (0.24 to 2.07)	−0.05 (−0.78 to 0.69)	−3.86 (−6.84 to −0.87)	0.78 (−0.62 to 2.18)	−0.12 (−0.29 to 0.05)	0.16 (−0.04 to 0.35)
**Time × treatment**							
	Week 2 × COMET-SSI	−0.33 (−0.79 to 0.14)	−0.08 (−0.49 to 0.34)	0.86 (−1.10 to 2.83)	−0.12 (−0.95 to 0.72)	0.03 (−0.07 to 0.12)	−0.09 −0.20 to 0.02)
	Week 4 × COMET-SSI	−0.39 (−0.90 to 0.12)	0.14 (−0.30 to 0.59)	1.46 (−0.66 to 3.58)	−0.12 (−1.02 to 0.77)	0.10 (0.00 to 0.21)	0.04 (−0.08 to 0.15)
	Week 8 × COMET-SSI	−0.22 (−0.79 to 0.36)	0.09 (−0.40 to 0.58)	0.92 (−1.44 to 3.27)	0.15 (−0.83 to 1.13)	0.04 (−0.08 to 0.16)	−0.10 (−0.22 to 0.02)

^a^Reference.

^b^COMET-SSI: Common Elements Toolbox–single-session intervention.

### ITT Sample Unimputed

Overall, there were no statistically significant differences between the COMET intervention (n=409) and WLC (n=419) in the short-term primary outcomes of depression and anxiety (Table 3). Not only were outcomes not significantly different between the groups but also the magnitude of the differences was quite small (Figure 2B). Similarly, there were no statistically or clinically significant differences between treatments in well-being, psychosocial functioning, or emotion regulation outcomes. The 8-week data showed an almost identical pattern of results, that is, there were no statistically significant differences between the treatment conditions. Multimedia Appendix 1 provides raw scores by treatment over time.

**Table 3:** Changes over time in depression, anxiety, well-being, functioning, and emotion regulation in 828 participants randomized 1:1 to a single-session intervention (n=409) versus a waiting list control (n=419), unimputed data.

Characteristics			Depression (Patient Health Questionnaire-9), B (95% CI)		Anxiety (Generalized Anxiety Disorder-7), B (95% CI)		Well-being (World Health Organization Well-Being Index-5), B (95% CI)		Functioning (Work and Social Adjustment Scale), B (95% CI)		Reappraisal (Emotion Regulation Questionnaire), B (95% CI)		Suppression (Emotion Regulation Questionnaire), B (95% CI)
Intercept			11.0 (10.3 to 11.6)		9.08 (8.56 to 9.61)		37.9 (35.7 to 40.0)		18.9 (17.9 to 19.9)		4.54 (4.42 to 4.66)		3.76 (3.62 to 3.90)
**Time**													
	Week 0	—^a^		—		—		—		—		—	
	Week 2	−0.95 (−1.34 to −0.57)		−0.92 (−1.27 to −0.58)		2.19 (0.46 to 3.91)		−1.83 (−2.53 to −1.12)		0.08 (0.00 to 0.16)		−0.03 (−0.12 to 0.06)	
	Week 4	−0.93 (−1.35 to −0.51)		−1.11 (−1.48 to −0.74)		1.77 (0.04 to 3.51)		−1.68 (−2.43 to −0.93)		−0.02 (−0.11 to 0.06)		−0.09 (−0.19 to 0.00)	
	Week 8	−1.46 (−1.93 to −0.99)		−1.33 (−1.72 to −0.94)		3.28 (1.55 to 5.00)		−2.03 (−2.84 to −1.23)		0.08 (−0.01 to 0.18)		−0.03 (−0.13 to 0.07)	
**Treatment**													
	Waiting list control	—		—		—		—		—		—	
	COMET-SSI^b^	1.17 (0.24 to 2.10)		−0.04 (−0.79 to 0.71)		−3.86 (−6.93 to −0.78)		0.78 (−0.64 to 2.20)		−0.12 (−0.30 to 0.05)		0.16 (−0.04 to 0.36)	
**Time × treatment**													
	Week 2 **×** COMET-SSI	−0.35 (−0.90 to 0.20)		−0.20 (−0.69 to 0.29)		0.77 (−1.67 to 3.22)		−0.16 (−1.15 to 0.84)		0.02 (−0.10 to 0.13)		−0.10 (−0.23 to 0.03)	
	Week 4 **×** COMET-SSI	−0.43 (−1.03 to 0.16)		0.09 (−0.43 to 0.61)		1.69 (−0.76 to 4.14)		−0.18 (−1.24 to 0.87)		0.13 (0.01 to 0.26)		0.05 (−0.08 to 0.19)	
	Week 8 **×** COMET-SSI	−0.19 (−0.85 to 0.48)		−0.01 (−0.58 to 0.55)		1.19 (−1.27 to 3.65)		0.17 (−0.98 to 1.32)		0.05 (−0.09 to 0.18)		−0.12 (−0.26 to 0.03)	

^a^Reference.

^b^COMET-SSI: Common Elements Toolbox–single-session intervention.

### Per-Protocol Analyses

The results with the “per-protocol” sample mirrored those of the imputed and imputed data sets, suggesting no statistically significant differences (WLC: n=417; COMET: n=379; Table 4). At week 2, the differences between the groups were small for depression (SMD −0.06, 95% CI −0.20 to 0.07), anxiety (SMD −0.05, 95% CI −0.18 to 0.09), well-being (SMD 0.02, 95% CI −0.12 to 0.16), functioning (SMD −0.04, 95% CI −0.18 to 0.09), cognitive reappraisal (SMD 0.03, 95% CI −0.11 to 0.16), or expressive suppression (SMD −0.08, 95% CI −0.22 to 0.06),

At week 8, the differences between the groups were small for depression (SMD −0.03, 95% CI −0.17 to 0.10), anxiety (SMD −0.01, 95% CI −0.15 to 0.12), well-being (SMD 0.06, 95% CI −0.08 to 0.19), functioning (SMD −0.02, 95% CI −0.16 to 0.12), cognitive reappraisal (SMD 0.04, 95% CI −0.10 to 0.18), and expressive suppression (SMD −0.07, 95% CI −0.21 to 0.06).

**Table 4:** Changes over time in depression, anxiety, well-being, functioning, and emotion regulation in 796 participants randomized 1:1 to a single-session intervention (n=417) versus a waiting list control (n=379), per-protocol sample.

Characteristics			Depression (Patient Health Questionnaire-9), B (95% CI)		Anxiety (Generalized Anxiety Disorder-7), B (95% CI)		Well-being (World Health Organization Well-Being Index-5), B (95% CI)		Functioning (Work and Social Adjustment Scale), B (95% CI)		Reappraisal (Emotion Regulation Questionnaire), B (95% CI)		Suppression (Emotion Regulation Questionnaire), B (95% CI)
Intercept			10.9 (10.3 to 11.6)		9.07 (8.55 to 9.60)		37.7 (35.6 to 39.9)		18.9 (17.9 to 19.9)		4.54 (4.41 to 4.66)		3.77 (3.63 to 3.91)
**Time**													
	Week 0	—^a^		—		—		—		—		—	
	Week 2	−0.94 (−1.32 to −0.55)		−0.90 (−1.24 to −0.55)		2.22 (0.59 to 3.84)		−1.78 (−2.47 to −1.09)		0.08 (0.00 to 0.16)		−0.03 (−0.12 to 0.06)	
	Week 4	−0.92 (−1.34 to −0.50)		−1.10 (−1.46 to −0.73)		1.93 (0.19 to 3.67)		−1.65 (−2.38 to −0.91)		−0.03 (−0.11 to 0.06)		−0.09 (−0.18 to 0.00)	
	Week 8	−1.44 (−1.91 to −0.98)		−1.33 (−1.72 to −0.93)		3.29 (1.40 to 5.19)		−1.97 (−2.77 to −1.18)		0.09 (−0.01 to 0.18)		−0.04 (−0.13 to 0.06)	
**Treatment**													
	Waiting list control	—		—		—		—		—		—	
	COMET-SSI^b^	1.14 (0.19 to 2.08)		−0.03 (−0.79 to 0.73)		−3.39 (−6.46 to −0.32)		1.10 (−0.35 to 2.55)		−0.13 (−0.30 to 0.05)		0.14 (−0.06 to 0.35)	
**Time × treatment**													
	Week 2 × COMET-SSI	−0.43 (−0.99 to 0.12)		−0.26 (−0.76 to 0.23)		0.45 (−1.90 to 2.79)		−0.45 (−1.45 to 0.55)		0.04 (−0.08 to 0.15)		−0.12 (−0.25 to 0.01)	
	Week 4 × COMET-SSI	−0.49 (−1.09 to 0.11)		0.11 (−0.41 to 0.64)		1.53 (−0.97 to 4.02)		−0.41 (−1.47 to 0.65)		0.16 (0.03 to 0.29)		0.08 (−0.06 to 0.21)	
	Week 8 × COMET-SSI	−0.21 (−0.89 to 0.47)		−0.06 (−0.63 to 0.51)		1.22 (−1.54 to 3.97)		−0.21 (−1.36 to 0.95)		0.05 (−0.09 to 0.19)		−0.11 (−0.25 to 0.03)	

^a^Reference.

^b^COMET-SSI: Common Elements Toolbox–single-session intervention.

### Sensitivity Analyses

To control for the possibility that our results were accounted for by inattentive respondents, we removed all participants who had failed even a single attention check, leaving us with a sample of 787 individuals (WLC: n=404; COMET-SSI: n=383). Across the 3 time points (weeks 2, 4, and 8), six outcomes (ie, depression, anxiety, well-being, functioning, cognitive reappraisal, and expressive suppression), and 3 data sets (ie, imputed, unimputed, and per-protocol), removing inattentive participants did not change the pattern of results (Multimedia Appendix 1). The largest intervention effect for depression was at week 4 in the per-protocol sample, representing a very small effect (B=−0.63, 95% CI −1.26, to −0.01; *P*=.046). Nonetheless, this was a very small effect, not corrected for multiple comparisons, which was inconsistent across the data sets and time points. Similarly, there was some evidence of a reduction in expressive suppression in COMET relative to WLC at week 2 (B=0.11, 95% CI −0.02 to 0.24; *P*=.09), but this was a small effect (SMD 0.07, 95% CI −0.07 to 0.21).

**Figure 3:**
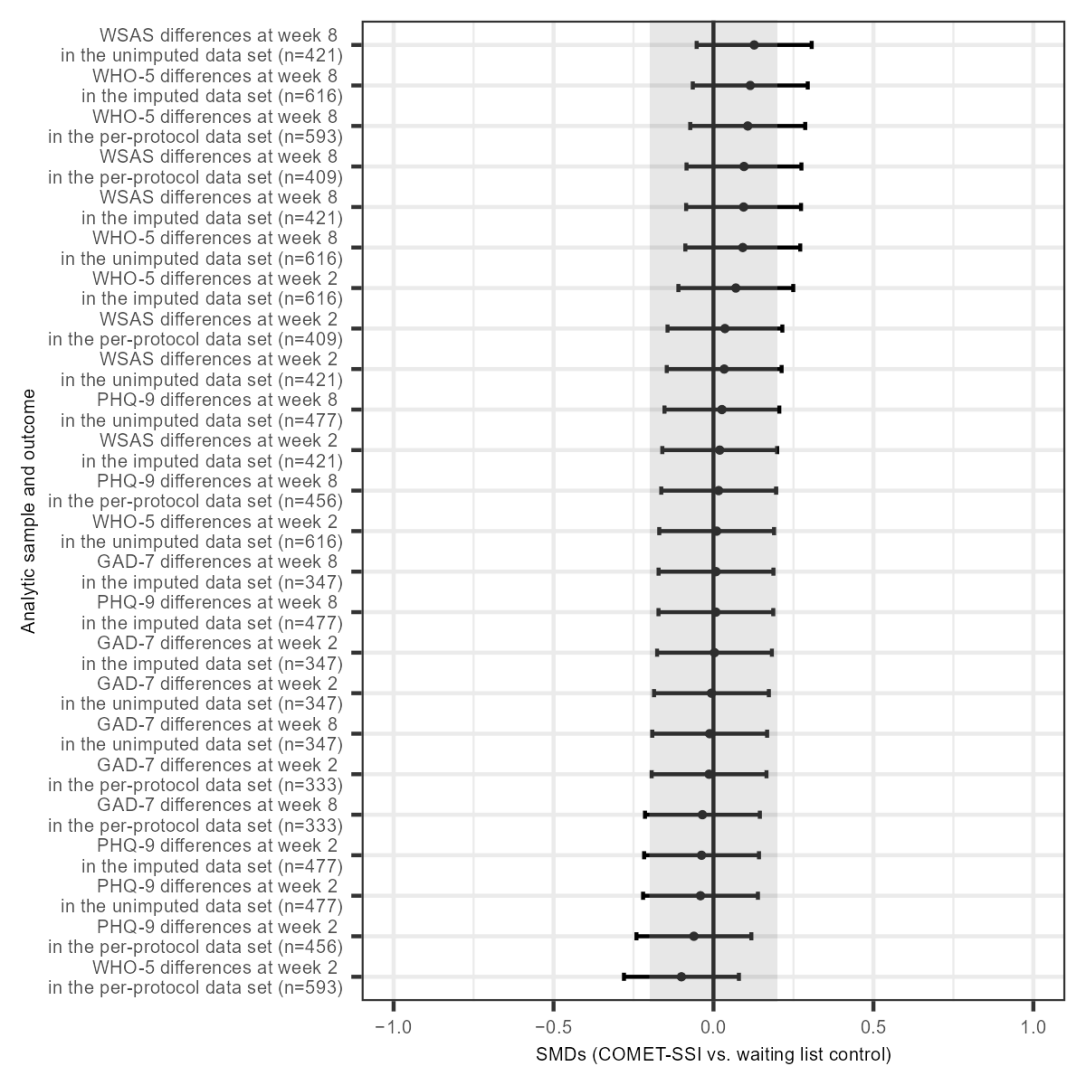
Standardized mean differences between the Common Elements Toolbox single-session intervention (COMET-SSI) and a waiting list control across different time points, in different data sets, for individuals scoring above the cutoff the different screening scales on the Patient Health Questionnaire-9 (PHQ-9), Generalized Anxiety Disorder-7 (GAD-7), Work and Social Adjustment Scale (WSAS), and World Health Organization Well-Being Index-5 (WHO-5).

In addition, given that we did not use severity on clinical screening scales as an entry criterion, it is possible that intervention effects may be observed in individuals with more severe psychopathologies. To address this possibility but balance multiple comparisons, we reran analyses for each of the subscales that can serve as screening scales for depression or anxiety (ie, the PHQ-9, GAD-7, WSAS, and WHO-5). For example, we reran the PHQ-9 analyses described earlier in the imputed, unimputed, and per-protocol samples in individuals with PHQ-9 scores ≥10. We reran all the GAD-7 analyses described earlier in the imputed, unimputed, and per-protocol samples in individuals with GAD-7 scores ≥10. The sample size for these sensitivity analyses ranged from 333 to 616. Assuming a *P* value of .05 (ie, not adjusting for multiple comparisons), we could detect effect sizes ranging from 0.22 to 0.31. Figure 3 shows a summary of effect sizes from the different measures (ie, PHQ-9, GAD-7, WSAS, and WHO-5) at different time points (ie, weeks 2 and 8) for the different data sets (ie, imputed, unimputed, and per-protocol). As shown in Figure 3, these effects were not statistically significant.

### Satisfaction Data

On average, participants reported high levels of satisfaction with COMET, suggesting they “agree” to “completely agree” that they are satisfied with the program (mean 3.11, SD 0.79).

## Discussion

### Principal Findings

We conducted an RCT to evaluate the efficacy of the COMET-SSI versus a WLC in Prolific participants. Our study was powered to detect small differences between the treatment conditions. The sample showed elevated levels of depression and anxiety, with most individuals being flagged on a screening scale for depression, anxiety, or poor functioning. However, our results did not suggest statistically significant differences between the COMET and WLC in any of the psychologically relevant outcomes we assessed, despite most individuals completing the intervention and even when focusing on individuals with more severe symptoms. Notably, participants in the COMET-SSI group were highly satisfied with the intervention, although they did not experience greater symptom improvement than participants in the WLC group. The correlation between participant satisfaction and treatment outcomes was known to be rather small [59].

It has been estimated that in the treatment of depression, effect sizes of approximately 0.24 may be deemed clinically significant [60]. Given that our study was powered to detect smaller differences, we must conclude that if the COMET-SSI has effects in paid web-based participants, the effects are so small that they are clinically questionable. Indeed, the 95% CIs for all of our effect size estimates mostly contained very small values (ie, +0.20 to −0.20). These results are consistent with the findings of Mullarkey et al [20] who did not find a statistically significant effect of an SSI on adult anxiety. Thus, our 2 studies suggest that paid web-based participants may be more treatment resistant than youth and college students. These results are in contrast with the results supporting the efficacy of DMHIs [11,14-16].

### Strengths and Limitations

Before interpreting our results, it is worth considering the aspects of our study design that may impose limitations on the inferences from these data. First, we recruited paid web-based participants for this study. Paid web-based participants have been noted as a population at high risk for depression [27]. We recruited participants if they had an “ongoing/diagnosed” mental health condition. Although the scores on the clinical screening scales suggest that most people would have met the criteria for a depressive or anxiety disorder if we had screened them (eg, with the PHQ-9), it is possible that we recruited many individuals who would not have benefited from treatment for depression or anxiety and may have benefited from other interventions (eg, substance use treatment and insomnia treatment). We did not recruit participants seeking help in routine care and instead provided treatment to individuals with mental health problems. However, Zhao et al [61] recently reported that psychological interventions can be effective, even when individuals are not seeking treatment. In addition, although Prolific is renowned for its data quality [62] and we reviewed participants’ responses for data quality, we have no way of confirming that respondents honestly engaged with the material after the intervention was completed. Although the COMET intervention asked participants to engage with the material by planning to use CBT and positive psychology principles, as in previous SSI studies, we did not measure adherence to the intervention outside its context of the intervention itself (eg, daily life). Our sample had a relatively high number of individuals who were identified as LGBTQ+. Other studies of unguided digital SSIs have reported high numbers of LGBTQ+ individuals [63,64], suggesting that in addition to a heightened risk of internalizing symptoms, LGBTQ+ individuals may have a greater willingness to engage with these treatments. Future studies should explore whether sexual orientation moderates the treatment outcomes and engagement. Finally, the typical limitations of web-based trials apply to this study, including the lack of concealment of intervention conditions to the participant, as well as the reliance on self-report measures.

This study had several strengths. First, we conducted a rather large study, which was well powered to detect small differences between treatments. This study is the largest assessment of the COMET-SSI and a very large trial as far as psychological interventions go [65]. Second, we preregistered our analytic plan and conducted the trial by concealing the intervention from the investigator and the analyst. Although we measured symptom outcomes of depression and anxiety, dubbed as the “vital signs” of mental health [66], we also assessed 2 important elements of quality of life—well-being and functioning—and 2 emotion regulation strategies—cognitive reappraisal and expressive suppression. In addition, consistent with calls for more generalizable depression research, we used relatively lax study entry criteria [67-69]. Finally, we used a multimethod approach to identify inattentive respondents, and our results suggested that most individuals were attentive.

Although SSIs have shown great promise in the treatment of adolescent internalizing distress [17] and in-person exposure-based SSIs have been very effective in the treatment of fear-based disorders in adults [70], less work exists on unguided SSIs in the treatment of adult internalizing distress. Notably, internalizing distress symptoms such as depression and generalized anxiety have been clinical problems for which it has been difficult to identify interventions that are more effective than controls [71,72]. Future work should explore the efficacy of SSIs for internalizing distress in other adult populations. Dobias et al [63] recently reported that an SSI focused on targeting self-hatred to reduce self-injurious behaviors was not significantly more effective than a control condition SSI in the study’s preregistered outcomes. Mullarkey et al [20] also reported that targeting perceived control over anxiety did not lead to improvements relative to the control condition. These findings, and ours, may suggest that there needs to be more care in the design of trials of SSIs in relation to the populations and clinical problems targeted. For some clinical problems and some populations, SSIs may be effective irrespective of the active mechanisms the interventions target. Alternatively, data-driven approaches may help to identify subgroups of individuals who preferentially respond to specific SSIs [5,6,73,74].

## Appendix

Multimedia Appendix 1 Sensitivity analyses.

Multimedia Appendix 2 CONSORT-eHEALTH checklist.

## Abbreviations

CBT: cognitive behavioral therapy
COMET: Common Elements Toolbox
DMHI: digital mental health intervention
ERQ: Emotion Regulation Questionnaire
GAD-7: Generalized Anxiety Disorder-7
ITT: intent-to-treat
LGBTQ+: lesbian, gay, bisexual, trans, queer, or other sexual minority
PHQ: Patient Health Questionnaire
PI: principal investigator
RCT: randomized controlled trial
SMD: standardized mean difference
SSI: single-session intervention
WHO-5: World Health Organization Well-Being Index-5
WLC: waiting list control
WSAS: Work and Social Adjustment Scale

## Data Availability

The data and preregistration are publicly available on the Open Science Foundation website [31,49].
