# Supplementary material for: Efficacy of an Unguided, Digital Single-Session Intervention for Internalizing Symptoms in Web-Based Workers: Randomized Controlled Trial
Source: J Med Internet Res. 2023 Jul 7;25:e45411. doi: 10.2196/45411 (PMC10362424; doi:10.2196/45411)
Supplement: Multimedia Appendix 1 [file jmir_v25i1e45411_app1.docx]

Appendix

Lorenzo Lorenzo-Luaces and Jacqueline Howard

2023-03-28

Table S1. Mean (and standard deviation) of outcomes over time, and by treatment condition, for 828 adults in an 8-week treatment study

|  | Waiting list (WLC) | | | | COMET-SSI | | | |
| --- | --- | --- | --- | --- | --- | --- | --- | --- |
| Characteristic | W0, N = 419^1^ | W2, N = 419^1^ | W4, N = 419^1^ | W8, N = 419^1^ | W0, N = 409^1^ | W2, N = 409^1^ | W4, N = 409^1^ | W8, N = 409^1^ |
| Depression (PHQ9) | 10.96 (6.64) | 9.66 (6.35) | 9.87 (6.41) | 9.33 (6.15) | 12.12 (6.76) | 10.96 (6.86) | 10.75 (7.00) | 10.53 (6.60) |
| Unknown | 1 | 81 | 89 | 84 | 1 | 80 | 78 | 89 |
| Anxiety (GAD-7) | 9.08 (5.50) | 7.89 (5.25) | 7.92 (5.43) | 7.61 (5.20) | 9.04 (5.45) | 8.02 (5.30) | 8.00 (5.48) | 7.79 (5.12) |
| Unknown | 2 | 81 | 89 | 82 | 0 | 80 | 77 | 94 |
| Well-being (WHO-5) | 37.88 (21.53) | 40.84 (22.68) | 39.99 (23.30) | 41.44 (23.09) | 34.02 (21.32) | 36.57 (23.38) | 37.50 (23.59) | 37.84 (22.67) |
| Unknown | 0 | 82 | 89 | 83 | 0 | 75 | 74 | 88 |
| Functioning (WSAS) | 18.93 (10.11) | 16.53 (10.29) | 16.94 (10.95) | 16.67 (10.39) | 19.74 (10.56) | 17.77 (11.01) | 17.65 (11.15) | 17.75 (11.23) |
| Unknown | 0 | 83 | 90 | 82 | 1 | 76 | 74 | 85 |
| Reappraisal (ERQ) | 4.54 (1.25) | 4.65 (1.29) | 4.55 (1.34) | 4.65 (1.32) | 4.42 (1.30) | 4.53 (1.29) | 4.57 (1.35) | 4.56 (1.33) |
| Unknown | 0 | 80 | 88 | 82 | 0 | 75 | 73 | 85 |
| Suppression (ERQ) | 3.76 (1.47) | 3.73 (1.51) | 3.64 (1.58) | 3.73 (1.60) | 3.92 (1.50) | 3.80 (1.51) | 3.88 (1.58) | 3.79 (1.54) |
| Unknown | 0 | 80 | 88 | 82 | 0 | 75 | 73 | 85 |
| ^1^Mean (SD) | | | | | | | | |
| PHQ-9 = Patient Health Questionnaire-9, GAD-7 = Generalized Anxiety Disorder-7, WHO-5 = World Health Organization Wellbeing Index-5, WSAS = Work and Social Adjustment Scale, ERQ = Emotion Regulation Questionnaire | | | | | | | | |

Table S2. Changes over time in depression, anxiety, well-being, functioning, and emotion regulation in participants randomized 1:1 to a single-session intervention vs. a waiting list control, imputed data removing inattentive responders (WLC = 404, COMET-SSI = 383).

|  | Depression (PHQ9) | | | Anxiety (GAD) | | | Well-being (WHO-5) | | | Functioning (WSAS) | | | Reappraisal (ERQ) | | | Suppression (ERQ) | | |
| --- | --- | --- | --- | --- | --- | --- | --- | --- | --- | --- | --- | --- | --- | --- | --- | --- | --- | --- |
| Characteristic | Beta | 95% CI^1^ | *P* | Beta | 95% CI^1^ | *P* | Beta | 95% CI^1^ | *P* | Beta | 95% CI^1^ | *P* | Beta | 95% CI^1^ | *P* | Beta | 95% CI^1^ | *P* |
| Time |  |  |  |  |  |  |  |  |  |  |  |  |  |  |  |  |  |  |
| W0 | — | — |  | — | — |  | — | — |  | — | — |  | — | — |  | — | — |  |
| W2 | -0.96 | -1.29, -0.63 | <0.001 | -0.94 | -1.23, -0.64 | <0.001 | 1.86 | 0.46, 3.26 | 0.009 | -1.83 | -2.42, -1.24 | <0.001 | 0.09 | 0.02, 0.16 | 0.010 | -0.01 | -0.09, 0.06 | 0.7 |
| W4 | -0.91 | -1.27, -0.54 | <0.001 | -1.05 | -1.36, -0.73 | <0.001 | 1.54 | 0.03, 3.05 | 0.046 | -1.62 | -2.26, -0.99 | <0.001 | 0.02 | -0.06, 0.09 | 0.7 | -0.07 | -0.15, 0.01 | 0.11 |
| W8 | -1.32 | -1.74, -0.90 | <0.001 | -1.27 | -1.62, -0.92 | <0.001 | 2.92 | 1.24, 4.60 | <0.001 | -2.01 | -2.70, -1.31 | <0.001 | 0.12 | 0.04, 0.20 | 0.005 | -0.03 | -0.11, 0.05 | 0.5 |
| Treatment |  |  |  |  |  |  |  |  |  |  |  |  |  |  |  |  |  |  |
| Waiting list (WLC) | — | — |  | — | — |  | — | — |  | — | — |  | — | — |  | — | — |  |
| COMET-SSI | 1.27 | 0.33, 2.20 | 0.008 | 0.08 | -0.68, 0.83 | 0.8 | -4.72 | -7.75, -1.69 | 0.002 | 0.97 | -0.46, 2.41 | 0.2 | -0.09 | -0.27, 0.08 | 0.3 | 0.18 | -0.02, 0.39 | 0.079 |
| Time * Treatment |  |  |  |  |  |  |  |  |  |  |  |  |  |  |  |  |  |  |
| W2 * COMET-SSI | -0.38 | -0.85, 0.10 | 0.12 | -0.12 | -0.54, 0.30 | 0.6 | 1.21 | -0.80, 3.21 | 0.2 | -0.10 | -0.95, 0.75 | 0.8 | 0.00 | -0.10, 0.10 | >0.9 | -0.11 | -0.22, 0.01 | 0.061 |
| W4 * COMET-SSI | -0.46 | -0.98, 0.07 | 0.087 | 0.07 | -0.38, 0.53 | 0.7 | 1.79 | -0.37, 3.96 | 0.10 | -0.12 | -1.03, 0.79 | 0.8 | 0.07 | -0.04, 0.17 | 0.2 | 0.01 | -0.10, 0.12 | 0.9 |
| W8 * COMET-SSI | -0.30 | -0.89, 0.30 | 0.3 | 0.03 | -0.48, 0.53 | >0.9 | 1.56 | -0.84, 3.97 | 0.2 | 0.21 | -0.79, 1.21 | 0.7 | 0.00 | -0.12, 0.12 | >0.9 | -0.12 | -0.23, 0.00 | 0.047 |
| ^1^CI = Confidence Interval | | | | | | | | | | | | | | | | | | |
| PHQ-9 = Patient Health Questionnaire-9, GAD-7 = Generalized Anxiety Disorder-7, WHO-5 = World Health Organization Wellbeing Index-5, WSAS = Work and Social Adjustment Scale, ERQ = Emotion Regulation Questionnaire | | | | | | | | | | | | | | | | | | |

Table S3. Changes over time in depression, anxiety, well-being, functioning, and emotion regulation in participants randomized 1:1 to a single-session intervention vs. a waiting list control, unimputed data, removing inattentive responders (WLC = 404, COMET-SSI = 383).

|  | Depression (PHQ9) | | | Anxiety (GAD) | | | Well-being (WHO-5) | | | Functioning (WSAS) | | | Reappraisal (ERQ) | | | Suppression (ERQ) | | |
| --- | --- | --- | --- | --- | --- | --- | --- | --- | --- | --- | --- | --- | --- | --- | --- | --- | --- | --- |
| Characteristic | Beta | 95% CI^1^ | *P* | Beta | 95% CI^1^ | *P* | Beta | 95% CI^1^ | *P* | Beta | 95% CI^1^ | *P* | Beta | 95% CI^1^ | *P* | Beta | 95% CI^1^ | *P* |
| Time |  |  |  |  |  |  |  |  |  |  |  |  |  |  |  |  |  |  |
| W0 | — | — |  | — | — |  | — | — |  | — | — |  | — | — |  | — | — |  |
| W2 | -0.97 | -1.37, -0.57 | <0.001 | -0.92 | -1.27, -0.57 | <0.001 | 2.20 | 0.53, 3.88 | 0.010 | -1.95 | -2.66, -1.23 | <0.001 | 0.10 | 0.01, 0.18 | 0.021 | -0.02 | -0.12, 0.07 | 0.7 |
| W4 | -0.94 | -1.37, -0.51 | <0.001 | -1.11 | -1.49, -0.74 | <0.001 | 1.87 | 0.08, 3.66 | 0.041 | -1.68 | -2.44, -0.92 | <0.001 | 0.00 | -0.09, 0.09 | >0.9 | -0.08 | -0.18, 0.01 | 0.083 |
| W8 | -1.40 | -1.89, -0.92 | <0.001 | -1.29 | -1.70, -0.89 | <0.001 | 3.26 | 1.31, 5.22 | 0.001 | -2.16 | -2.98, -1.33 | <0.001 | 0.11 | 0.01, 0.20 | 0.033 | -0.03 | -0.13, 0.06 | 0.5 |
| Treatment |  |  |  |  |  |  |  |  |  |  |  |  |  |  |  |  |  |  |
| Waiting list (WLC) | — | — |  | — | — |  | — | — |  | — | — |  | — | — |  | — | — |  |
| COMET-SSI | 1.35 | 0.39, 2.31 | 0.006 | 0.12 | -0.65, 0.89 | 0.8 | -4.97 | -8.08, -1.87 | 0.002 | 0.99 | -0.48, 2.47 | 0.2 | -0.09 | -0.27, 0.08 | 0.3 | 0.19 | -0.02, 0.40 | 0.083 |
| Time * Treatment |  |  |  |  |  |  |  |  |  |  |  |  |  |  |  |  |  |  |
| W2 * COMET-SSI | -0.42 | -0.99, 0.14 | 0.14 | -0.23 | -0.73, 0.28 | 0.4 | 0.96 | -1.43, 3.36 | 0.4 | -0.04 | -1.06, 0.97 | >0.9 | -0.01 | -0.13, 0.11 | 0.8 | -0.12 | -0.25, 0.02 | 0.095 |
| W4 * COMET-SSI | -0.56 | -1.17, 0.06 | 0.077 | 0.01 | -0.53, 0.54 | >0.9 | 1.87 | -0.69, 4.42 | 0.2 | -0.12 | -1.20, 0.96 | 0.8 | 0.08 | -0.05, 0.21 | 0.2 | 0.03 | -0.11, 0.17 | 0.7 |
| W8 * COMET-SSI | -0.32 | -1.01, 0.37 | 0.4 | -0.07 | -0.65, 0.52 | 0.8 | 1.63 | -1.18, 4.45 | 0.3 | 0.34 | -0.85, 1.52 | 0.6 | 0.00 | -0.14, 0.14 | >0.9 | -0.13 | -0.26, 0.01 | 0.069 |
| ^1^CI = Confidence Interval | | | | | | | | | | | | | | | | | | |
| PHQ-9 = Patient Health Questionnaire-9, GAD-7 = Generalized Anxiety Disorder-7, WHO-5 = World Health Organization Wellbeing Index-5, WSAS = Work and Social Adjustment Scale, ERQ = Emotion Regulation Questionnaire | | | | | | | | | | | | | | | | | | |

Table S4. Changes over time in depression, anxiety, well-being, functioning, and emotion regulation in participants randomized 1:1 to a single-session intervention vs. a waiting list control, per protocol sample, removing inattentive responders (WLC = 404, COMET-SSI = 383).

|  | Depression (PHQ9) | | | Anxiety (GAD) | | | Well-being (WHO-5) | | | Functioning (WSAS) | | | Reappraisal (ERQ) | | | Suppression (ERQ) | | |
| --- | --- | --- | --- | --- | --- | --- | --- | --- | --- | --- | --- | --- | --- | --- | --- | --- | --- | --- |
| Characteristic | Beta | 95% CI^1^ | *P* | Beta | 95% CI^1^ | *P* | Beta | 95% CI^1^ | *P* | Beta | 95% CI^1^ | *P* | Beta | 95% CI^1^ | *P* | Beta | 95% CI^1^ | *P* |
| Time |  |  |  |  |  |  |  |  |  |  |  |  |  |  |  |  |  |  |
| W0 | — | — |  | — | — |  | — | — |  | — | — |  | — | — |  | — | — |  |
| W2 | -0.96 | -1.35, -0.56 | <0.001 | -0.89 | -1.26, -0.53 | <0.001 | 2.22 | 0.46, 3.98 | 0.013 | -1.90 | -2.60, -1.20 | <0.001 | 0.10 | 0.01, 0.18 | 0.024 | -0.02 | -0.11, 0.07 | 0.6 |
| W4 | -0.93 | -1.36, -0.50 | <0.001 | -1.10 | -1.47, -0.73 | <0.001 | 2.04 | 0.27, 3.82 | 0.024 | -1.65 | -2.40, -0.90 | <0.001 | 0.00 | -0.09, 0.09 | >0.9 | -0.08 | -0.18, 0.01 | 0.082 |
| W8 | -1.39 | -1.87, -0.91 | <0.001 | -1.28 | -1.65, -0.91 | <0.001 | 3.23 | 1.48, 4.99 | <0.001 | -2.09 | -2.90, -1.28 | <0.001 | 0.11 | 0.01, 0.20 | 0.033 | -0.03 | -0.14, 0.07 | 0.5 |
| Treatment |  |  |  |  |  |  |  |  |  |  |  |  |  |  |  |  |  |  |
| Waiting list (WLC) | — | — |  | — | — |  | — | — |  | — | — |  | — | — |  | — | — |  |
| COMET-SSI | 1.35 | 0.37, 2.33 | 0.007 | 0.16 | -0.60, 0.93 | 0.7 | -4.64 | -7.85, -1.44 | 0.005 | 1.39 | -0.12, 2.89 | 0.071 | -0.10 | -0.28, 0.08 | 0.3 | 0.18 | -0.03, 0.40 | 0.086 |
| Time * Treatment |  |  |  |  |  |  |  |  |  |  |  |  |  |  |  |  |  |  |
| W2 * COMET-SSI | -0.52 | -1.09, 0.06 | 0.077 | -0.31 | -0.84, 0.23 | 0.3 | 0.60 | -1.96, 3.16 | 0.6 | -0.32 | -1.34, 0.70 | 0.5 | 0.01 | -0.11, 0.13 | >0.9 | -0.14 | -0.27, -0.01 | 0.039 |
| W4 * COMET-SSI | -0.63 | -1.26, -0.01 | 0.046 | 0.03 | -0.51, 0.57 | >0.9 | 1.72 | -0.85, 4.28 | 0.2 | -0.38 | -1.46, 0.70 | 0.5 | 0.11 | -0.02, 0.24 | 0.089 | 0.05 | -0.09, 0.19 | 0.5 |
| W8 * COMET-SSI | -0.36 | -1.07, 0.35 | 0.3 | -0.13 | -0.67, 0.41 | 0.6 | 1.54 | -1.03, 4.12 | 0.2 | -0.09 | -1.27, 1.10 | 0.9 | 0.01 | -0.13, 0.15 | 0.9 | -0.12 | -0.27, 0.03 | 0.11 |
| ^1^CI = Confidence Interval | | | | | | | | | | | | | | | | | | |
| PHQ-9 = Patient Health Questionnaire-9, GAD-7 = Generalized Anxiety Disorder-7, WHO-5 = World Health Organization Wellbeing Index-5, WSAS = Work and Social Adjustment Scale, ERQ = Emotion Regulation Questionnaire | | | | | | | | | | | | | | | | | | |
